# Deep-learning-powered photonic analog-to-digital conversion

**DOI:** 10.1038/s41377-019-0176-4

**Published:** 2019-07-17

**Authors:** Shaofu Xu, Xiuting Zou, Bowen Ma, Jianping Chen, Lei Yu, Weiwen Zou

**Affiliations:** State Key Laboratory of Advanced Optical Communication Systems and Networks, Intelligent Microwave Lightwave Integration Innovation Center (iMLic), Department of Electronic Engineering, Shanghai Jiao Tong University, 200240 Shanghai, China

**Keywords:** Microwave photonics, Optoelectronic devices and components

## Abstract

Analog-to-digital converters (ADCs) must be high speed, broadband, and accurate for the development of modern information systems, such as radar, imaging, and communications systems; photonic technologies are regarded as promising technologies for realizing these advanced requirements. Here, we present a deep-learning-powered photonic ADC architecture that simultaneously exploits the advantages of electronics and photonics and overcomes the bottlenecks of the two technologies, thereby overcoming the ADC tradeoff among speed, bandwidth, and accuracy. Via supervised training, the adopted deep neural networks learn the patterns of photonic system defects and recover the distorted data, thereby maintaining the high quality of the electronic quantized data succinctly and adaptively. The numerical and experimental results demonstrate that the proposed architecture outperforms state-of-the-art ADCs with developable high throughput; hence, deep learning performs well in photonic ADC systems. We anticipate that the proposed architecture will inspire future high-performance photonic ADC design and provide opportunities for substantial performance enhancement for the next-generation information systems.

## Machine learning: Neural networks could help herald next-generation information systems

Scientists have used deep learning technologies to improve the performance of photonic analogue-to-digital converters (ADCs), opening the door to advanced information systems. Modern information processing technologies, such as radar, imaging, and communications systems, require ADCs that are high speed, accurate and have broadband capacity. Although electronic ADCs offer high speed and accuracy, their bandwidth is limited, hindering the development of more advanced information technologies. Now, Weiwen Zou and colleagues from Shanghai Jiao Tong University in China have overcome this limitation by using deep learning technologies to improve the performance of photonic ADCs. By combining the data recovery capabilities of neural networks with the technical advantages of electronics and photonics technologies, the team created a photonic ADC that overcomes the limitations on bandwidth, laying the foundations for next-generation information systems, including ultra-wideband radars and high-resolution microwave imaging.

## Introduction

Next-generation information systems, such as radar, imaging, and communications systems, are aimed at realizing high operation frequencies and broad bandwidths, and require analog-to-digital converters (ADCs) with high sampling rate, broadband coverage, and sufficient accuracy^1–3^. Traditionally, in modern information systems, electronic analog-to-digital conversion methods have supported high-accuracy quantization and operational stability due to the mature manufacturing of electronic components; nevertheless, their bandwidth limitations and high timing jitter hinder the development of electronic methods toward broadband high-accuracy ADCs for next-generation information systems^3–7^. Facilitated by photonic technologies, the bottlenecks of bandwidth limitations and timing jitter are elegantly overcome^4^. However, since the imperfect properties and setups of photonic components give rise to system defects and can deteriorate the performance of ADCs^4,8,9^, designing an advanced ADC architecture remains challenging.

Recently, deep learning technologies^10^ have made substantial advances in a variety of artificial intelligence applications, such as computer vision^11,12^, medical diagnosis^13^, and gaming^14^. By constructing multiple layers of neurons and applying appropriate training methods, data from images, audio, and video can be automatically extracted with representations to be used in the inference of unknown data. Data recovery and reconstruction tasks, including speech enhancement^15^, image denoising^16^, and reconstruction^17,18^, are well accomplished with convolutional neural networks (CNNs, neural networks based on convolutional filters), thereby demonstrating the ability of deep neural networks to learn the model of data contamination and distortion and to output the recovered data. Therefore, it is believed that machine learning technologies, including deep learning, can offer substantial power for photonic applications^19,20^.

**Fig. 1:**
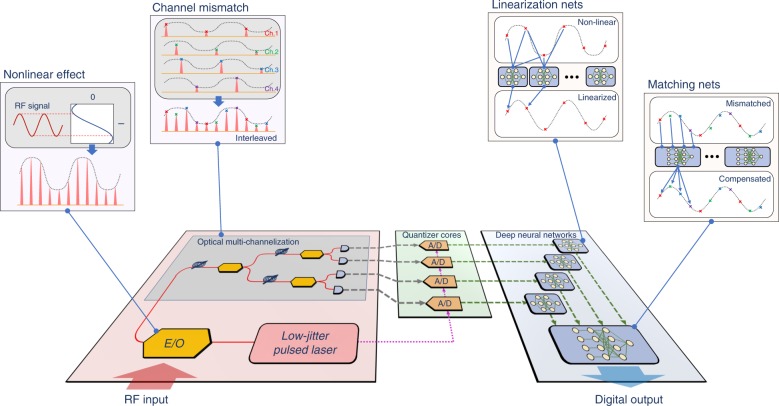
Schematic representations of the DL-PADC architecture. Three cascaded parts (a photonic front end, electronic quantization, and deep learning data recovery) are illustrated with various background colors. In the photonic front-end, E/O provides a broad bandwidth for receiving RF but introduces nonlinearity into the system due to its sinusoidal-like transfer function. Illustrated in the subplot is the nonlinear effect: a standard sine RF signal that will be distorted by the nonlinear transfer function, thereby distorting the sampling pulses. In addition, to the best of our knowledge, all the reported methods of multichannelization (a two-stage optical time-divided demultiplexing method is depicted as an example) introduce channel mismatch (see the subplot of channel mismatch). To ensure the high accuracy of electronic quantizers, not only should the low-jitter pulsed laser offer a high-quality clock, but the distorted signal should also be recovered. Cascaded after each electronic quantizer, the neural network linearization nets recover the nonlinearity distortions via time-domain convolutions; the matching nets receive all channels’ linearized output data and interleave them with the channel mismatch compensated

**Fig. 2:**
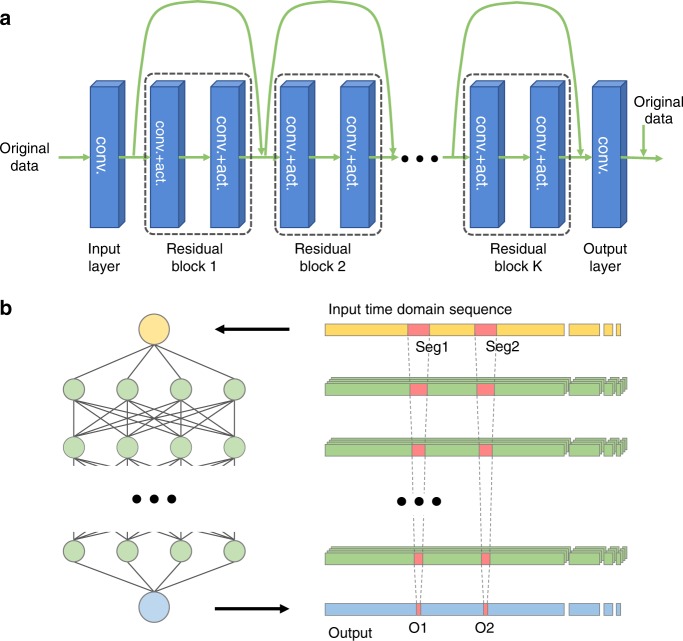
Basic model of linearization nets and matching nets. **a** The adopted residual-on-residual learning model. The input layer accepts the original data and converts them to multiple feature channels via convolutions. From the second layer, each layer is constructed with convolutions and nonlinear activation functions (rectified linear units, which are denoted as ReLUs, in this work). In addition, the two convolutional layers compose a residual block. At the end of each residual block, the data are combined with those ahead of the residual block to complete a residual short-cut. After several residual blocks, the output layer merges data from multiple feature channels and adds them to the original data. Conv. represents convolution and act. denotes nonlinear activation. **b** A schematic diagram of the data manipulation in fully convolutional neural networks, in which all links are convolutions. Because convolution requires only several input values to yield results, a single output value (O1 or O2) is only influenced by several values from the previous layer (red block). If we inspect the neural network in reverse, the value of O1 is only influenced by a small segment in the input sequence (Seg1) and all other values are irrelevant. O2 and Seg2 are similar (detailed in the Suppl.)

By taking advantage of data recovery via deep learning technology, we present an architecture for constructing high-performance ADCs. As illustrated in Fig. 1, the deep-learning-powered photonic analog-to-digital conversion (DL-PADC) architecture is composed of three main cascaded parts: a photonic front-end^4^, electronic quantization, and deep learning data recovery. In the photonic front-end, a low-jitter pulsed laser source^8,9^ provides the sampling optical pulse train and the precise quantization clock, thereby ensuring low noise from the source. An electrooptic modulator (E/O) subsequently provides broadband radio frequency (RF) reception by incorporating the photonic advantage in terms of signal bandwidth. Via optical multichannelization^21^, the sampling speed in each channel is lowered for compatibility with the electronic quantization. Driven by the precise quantization clock from the optical source, electronic quantizers are exploited with their high quantization accuracy. In practice, the defects in the photonic front-end can pervade the quantized data; hence, the deep learning data recovery realizes distortion elimination of the quantized data, which is essential for overcoming the tradeoff among bandwidth, sampling rate, and accuracy for traditional ADCs. Deep learning data recovery includes two steps with two functional neural networks: “linearization nets” and “matching nets.” The former executes nonlinearity elimination and the latter interleave data in multiplexed channels with channel mismatch compensation. Unlike the traditional dual-balanced linearization method^7^, the linearization nets require no complicated setups or miscellaneous data processing steps. In addition, the matching nets accomplish the interleaving via time-domain representations. Because time-domain representations avoid the problems of data length variation and spectrum aliasing, matching nets are more effective than spectral analysis algorithms, which are adopted in state-of-the-art mismatch compensation schemes^8,9^. In Fig. 2, we illustrate the basic models of the two types of neural networks (linearization nets and matching nets) that are adopted in the experimental implementation. As shown in Fig. 2a, the model is implemented with cascading convolutional layers via residual learning schemes^22^ and the output of the last layer is summed with the input data^23^. Figure 2b presents a schematic diagram of convolutions in the neural networks and explains why these fully convolutional neural networks are immune to input length variation and spectrum aliasing. A single value of the output depends only on a small segment from the input sequence, namely, the convolutional windows are trained to learn the local relations of the output and the corresponding input segment (detailed in the Suppl.). If the input length varies, only the number of segments changes and the neural networks still map these segments to outputs correctly. In addition, because the input and output sequences are specified in the time domain, the limitations of spectral analysis methods can be avoided in these neural networks.

## Results and discussion

**Fig. 3:**
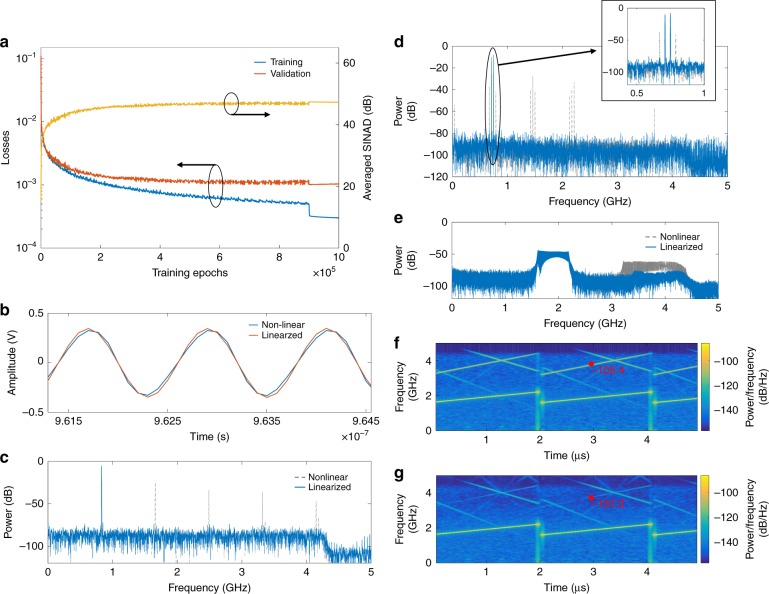
Results for validation of linearization nets. **a** The performance of neural networks under training. The training loss and validation loss decrease as the number of training epochs increases. The training loss is obtained from the training set by calculating the absolute error between the network output and the reference data. The validation loss is the absolute error in the validation set, which is not overlapped with the training set. To facilitate understanding of the linearization performance, the yellow curve represents the average SINAD in the validation set. **b**, **c** Examples of nonlinear distortion elimination in the time domain and the frequency domain, respectively. The test sine signal is 833 MHz, ± 0.36 V. **d** An example of neural network applicability with untrained dual-tone signals. The gray dashed curve and the blue curve represent the data before and after the linearization nets, respectively. The dual-tone signals are of frequencies 712 and 752 MHz. **e**–**g** An example of neural network applicability with untrained LFM signals. The frequency spectrum and the STFT spectra are presented to illustrate the elimination of distortions. We mark the values at the same location in the upper (before) and lower (after) STFT spectra. The LFM signal is in the 1.60–2.20-GHz range

Based on the neural network model and the DL-PADC architecture, we constructed experimentally a two-channel 20-Gsample/s photonic ADC for a proof-of-concept demonstration (detailed in “Methods”). Linearization nets and matching nets were constructed and trained with distorted data and their corresponding reference data (the neural network implementation, data acquisition, processing, and training procedures are detailed in “Methods”). Figure 3 plots the performances of linearization nets with various waveforms. During training, untrained sine data with various frequencies and amplitudes were used to evaluate the inference performance of the neural networks (i.e., validation). Figure 3a presents the variations of the training loss and the validation loss as the number of training epochs increases. Here, the loss represents the absolute error between the network output and the reference data; the network output approaches the reference data as the loss decreases. The training loss is calculated as an average over the data in the training set and the valid loss is calculated as an average over the validation set. The losses decrease as the number of training epochs increases and converge to steady levels. To facilitate comprehension, the average signal-to-noise and distortion ratio (SINAD) is also calculated for the validation set. It converges to ~47 dB; hence, linearization nets are a viable approach for the nonlinearity correction of untrained data that are spread over the whole spectrum. As an example, an untrained signal in the time and frequency domains before and after the linearization nets is shown in Fig. 3b, c. The E/O-distorted waveform is corrected to a sine signal. In the frequency domain, the harmonics that are due to the E/O nonlinearity have been eliminated. To evaluate the broader applicability of linearization nets with other sine-like signals, we used dual-tone signals and linear frequency modulated (LFM) signals to evaluate networks that were only trained by sine signals. As shown in Fig. 3d, prior to linearization, dual-tone signals are distorted by E/O to produce a series of distortions on the frequency spectrum; these distortions are effectively eliminated by the trained linearization nets. The results demonstrate that the linearization nets can substantially extend the spurious-free dynamic range (SFDR) of the received signal amplitude, thereby ensuring high accuracy of the DL-PADC. In the spectrum that is shown in Fig. 3e, the distortions of the LFM signals are suppressed. In the short-time Fourier transformation (STFT) spectrum (Fig. 3f, g), we realized an ~26 dB improvement of the signal-to-distortion ratio after the neural networks. The applied LFM signal source (an arbitrary waveform generator) has an effective number of bits (ENOB) accuracy of ~6; hence, the noise and distortions in the LFM signal itself are relatively high, thereby degrading the effectiveness of the neural networks. More complete test results for linearization nets are presented in Supplementary Figs. S3 and S4, where the results demonstrate the reliability of linearization nets in nonlinearity correction.

**Fig. 4:**
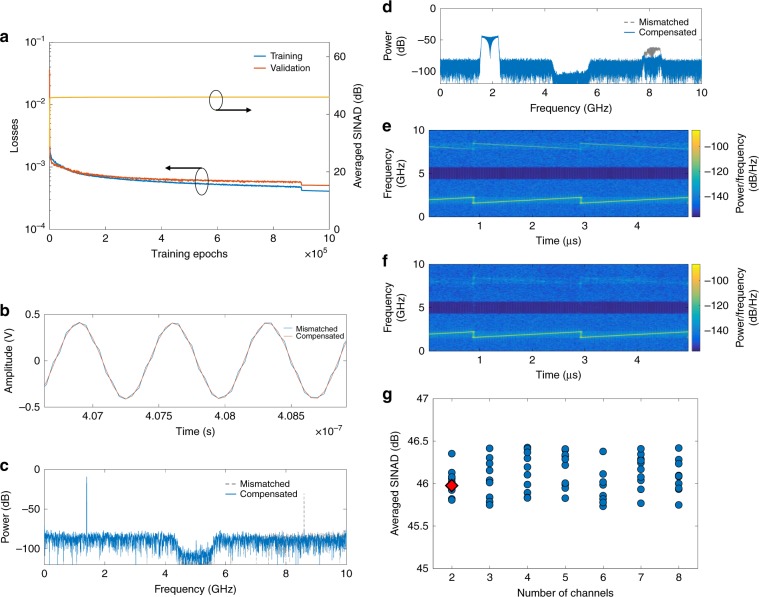
Results for the validation of matching nets. **a** The losses decrease as the number of training epochs increases. The yellow curve represents the averaged SINAD in the validation set. **b**, **c** Examples of channel mismatch compensation with matching nets in the time domain and the frequency domain, respectively. The test signal is 1.403 GHz and ±0.42 V. **d**–**f** The frequency spectrum and STFT spectra are shown to illustrate the neural network’s applicability with untrained LFM signals. The signal is in the 1.60–2.20-GHz range. **g** Simulation results of the expandability of matching nets for a larger number of channels. We considered various numbers of channels (2–8) and various mismatch degrees (referring to the mismatch degree in our experiment). For every combination of number of channels and mismatch degree, we conducted 0.5 million training epochs and specified the average SINAD in the figure. The red diamond denotes the experimental result

Figure 4 shows the performance of the matching nets. We consider each reference data of the linearization nets as a single input of the matching nets and train the network with reference interleaved data. Figure 4a shows the results of training the matching nets. As the number of epochs increases, the training and validation losses decrease and converge to steady levels and the average SINAD approaches the noise-floor-limited level, namely, ~46 dB. An example of the sine signal is presented in Fig. 4b, c. In the time-domain plot, channel mismatch produces errors on the interleaved data and incorporates the mismatch distortions into the frequency spectrum; the errors are corrected and the mismatch distortions are compensated effectively with matching nets. Furthermore, the matching nets can realize channel mismatch compensation of broadband signals. Figure 4d–f presents an example of the compensation of a mismatch-distorted LFM signal. On the right side of the frequency spectrum is the broadband distortion that was introduced by the channel mismatch. The matching nets eliminate it effectively, as shown in the following STFT spectra. Since the number of channels determines the sampling rate product and the electronic burden release, the matching nets should also be compatible with multichannel data interleaving. To ensure the expandability of the constructed matching nets, simulations were conducted with various numbers of channels (detailed in “Methods”). For various numbers of channels and randomly selected mismatch degrees, we trained the matching nets to interleave mismatched data; the average SINAD in the validation set converges at ~46 dB (Fig. 4g); hence, the matching nets can adapt to various numbers of channels and various mismatch degrees. These results, together with additional test results (Supplementary Figs. S5 and S6), validate the matching nets in channel mismatch compensation.

**Fig. 5:**
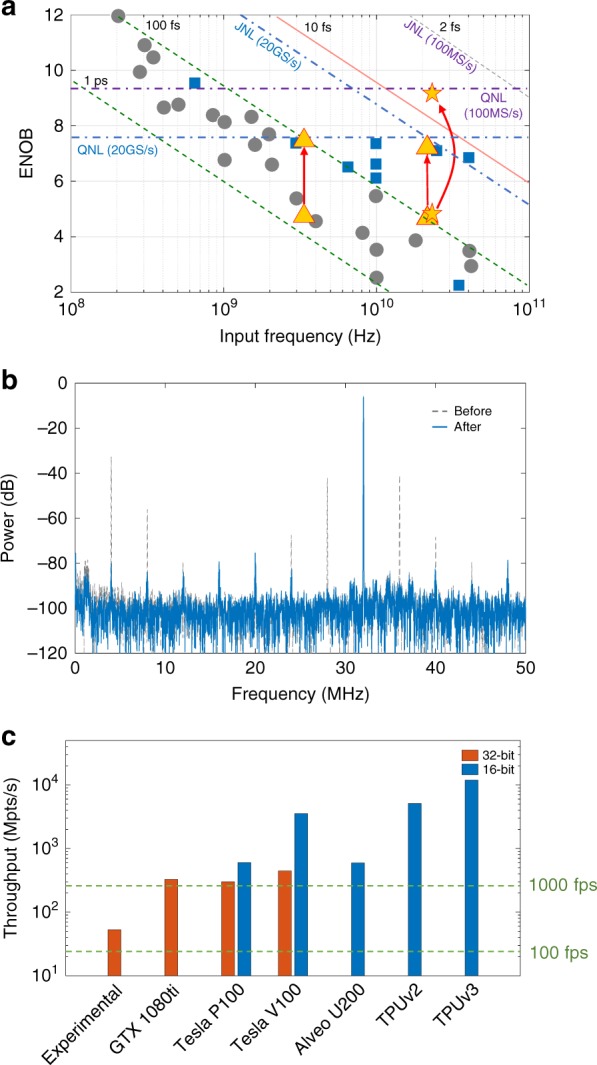
Performance characterization of the proposed DL-PADC architecture. **a** A Walden plot of performances of state-of-the-art ADC systems (data sampled from ref. ^3^). The gray dots represent the performances of electronic ADCs and the blue rectangles the photonics-assisted ADC performances. The yellow triangles represent the performance improvements of the experimental setup for ADC before and after the application of deep learning (the signal frequencies are 3.44 and 21.13 GHz, respectively). The yellow stars represent the ENOB results for the additional experiment with a 100-MHz MLL and a 100-MS/s data acquisition board (the signal frequency is 23.332 GHz). Reference lines that represent the theoretical values (evaluated in “Methods”) of the quantization noise limitation (QNL) and jitter noise limitation (JNL) are marked. **b** The frequency spectra before and after nonlinearity cancellation by the linearization nets using the MLL and data acquisition board in the experimental setup. The input frequency is 23.332 GHz. **c** The throughput evaluation of the neural networks based on various commercial deep learning accelerators. The experiments are conducted using a dual-GPU platform. The other throughput results are theoretically evaluated according to the officially declared performances in terms of floating-point operations per second (FLOPS). Performances that are based on two data types (32-bit or 16-bit floating-point) are compared using two color bars. The fps of the neural network output is also evaluated. The input data cache in each frame is assumed as 256 kpts and levels of 100 ftps and 1000 fps are indicated (see “Methods” for the evaluation details)

As the effectiveness of the neural networks has been completely demonstrated, we characterize the performance enhancement of the experimental 20-Gsamples/s photonic ADC setup and compare it with state-of-the-art commercial and in-lab ADCs by using the Walden plot. We evaluate sine signals with frequencies of 3.44 and 21.13 GHz using the experimental setup. Before the test signals are sampled and quantized, the training procedure is executed with the training set that is described above. In principle, the 21.13-GHz signal will be subsampled to 1.13 GHz so that the trained neural networks can adapt when directly sampling high-frequency signals. In Fig. 5a, two results are presented for each test signal: prior to data recovery by the neural networks, the DL-PADC performs 4.66 ENOB with an input frequency of 3.44 GHz and 4.53 ENOB with 21.13 GHz. After two cascaded steps of data recovery, the results reach 7.28 ENOB with an input frequency of 3.44 GHz and 7.07 ENOB with 21.13 GHz. The accuracy performance does not surpass those of the state-of-the-art ADCs because it is realized with inferior electronic quantization (the oscilloscope), of which the quantization noise heavily limits the accuracy enhancement. To demonstrate the ultimate accuracy of the neural networks, we conducted an additional experiment with a 100-MHz mode-locked laser (MLL) with nominal 2-fs timing jitter and a 100-MS/s high-accuracy data acquisition board (detailed in “Methods”). Although the sampling rate is low, this experimental setup provides an ultralow noise level, thereby demonstrating the performance of the neural networks in terms of accuracy. We evaluated the accuracy performance of the linearization nets, which did not differ substantially from the performance of the matching nets. The ENOB results are also shown in Fig. 5a. With the elimination of nonlinear distortions, the ENOB has been enhanced from 4.57 to 9.24 with an input frequency of 23.332 GHz. Referring to the quantization noise and jitter noise limitations, the performance of the DL-PADC could closely approach the theoretical limitations for high-frequency RF signals. Figure 5b shows the spectrum of the linearized 23.332-GHz signal, which demonstrates that nonlinear distortions are effectively eliminated and the SFDR is substantially enlarged. By testing the signals over the whole frequency range, the SFDR is characterized above 68 dB and is 71 dB on average (the ENOB and SFDR characterizations are described in “Methods”).

Figure 5c illustrates the throughput evaluations of the neural networks on various computing platforms (The details of throughput evaluations could be found in “Methods”). In the experiments, two paralleled GTX 1080ti graphic processing units (GPUs) are adopted and the throughput of the neural networks is 52.92 mega-points per second (Mpts/s) in 32-bit float. Due to the unoptimized codes and resource management, the experimental result and the nominal performance of GTX 1080ti differ. Moreover, we evaluate the throughputs when the neural networks are implemented on the state-of-the-art commercial deep learning accelerators and observe substantial enhancement if faster processers are applied. For example, the throughput on Google TPU v3 is evaluated to be 11930 Mpts/s theoretically. Compared with the high sampling rate of DL-PADC architecture, namely, several tens of GS/s, the throughput of the neural networks appears to be lower. However, in practical applications, the signal length is quantified in frames. Assuming the input cache in each frame is 256 kpts, the neural networks can output the recovered data at ~200 frames per second (fps) using the experimental setup. Furthermore, due to the recent progress in deep learning accelerators via electronic^24–26^ and optical^27,28^ schemes, the throughput of the data recovery neural networks could increase substantially in the near future.

## Conclusions

The proposed DL-PADC architecture combines the technical advantages of electronic, photonic, and neural networks for the first time. By deploying fully convolutional neural networks for effective data recovery, this architecture provides a succinct and developable approach for overcoming the existing bottlenecks of ADCs. As is demonstrated in the numerical and experimental results, DL-PADC outperforms state-of-the-art commercial and in-lab ADCs and is compatible with further performance enhancement in terms of accuracy and neural network throughputs. We anticipate that the proposed architecture could inspire the use of deep learning technology for photonic ADC refinement and enable further development of high-performance ADCs. In addition, with the augmentation or alteration of datasets (detailed in Suppl.), the proposed architecture could essentially pave the way for next-generation information systems, especially in high-frequency and broadband scenarios such as ultra-wideband radars, high-resolution microwave imaging, and advanced RF measurement.

## Materials and methods

### Experimental setup of the 20-GS/s photonic ADC

Based on the proposed DL-PADC architecture, we set up a two-channel 20-GS/s photonic ADC for validation (the experimental setup is shown in Supplementary Fig. S2). We implemented the photonic front-end with an actively mode-locked laser (AMLL, CALMAR PSL-10-TT), a microwave generator (MG1, KEYSIGHT E8257D), a Mach–Zehnder modulator (MZM, PHOTLINE MXAN-LN-40), and a two-channel time-division demultiplexer. Driven by the MG1 at a frequency of 20 GHz, the AMLL emitted optical pulses at a 20-GHz repetition rate. As a reference, the measured timing jitter of the AMLL output optical pulse was ~26.5 fs. The MZM adopted had a bandwidth of 40 GHz, thereby guaranteeing the reception of high-frequency broadband signals. In the MZM, the optical pulse train from the AMLL was amplitude-modulated by the signal to be sampled; therefore, the signal was sampled with a fixed interval. The two-channel time-divided demultiplexer consisted of a tunable delay line (TDL, General Photonics MDL-002) with a tuning accuracy of 1 ps, a dual-output MZM (DOMZM, PHOTLINE AX-1 × 2–0MsSS-20-SFU-LV) of low quadrature voltage $V_\pi = 3.5{\rm{V}}$, and two identical custom-built PDs of 10-GHz bandwidth. For demultiplexing the optical pulse train into two channels, the custom-built frequency divider transferred the 20-GHz signal from the MG1 to 10 GHz and drove the DOMZM. The DOMZM was biased at its quadrature point and the driving 10-GHz signal was adjusted to match the full *V*_*π*_ of the DOMZM. Subsequently, we adjusted the TDL to allow one optical pulse of two adjacent pulses to pass through the DOMZM at its maximal transmission rate and allow the other pulse to pass through the MZM at its minimal transmission rate. Therefore, the optical pulse train was demultiplexed into two channels. To evaluate the effectiveness of the demultiplexer, we used a 50-GHz PD (u2t XPDV2150R) and a sampling oscilloscope (KEYSIGHT DCA-X 86100D) to test the demultiplexed optical pulses. During the electronic quantization, a multichannel real-time oscilloscope (OSC, KEYSIGHT DSO-S 804 A) was adopted as the quantizer; it had a 10-GS/s sampling speed and four channels. As a reference, we measured the ENOB of the OSC at 7.4 maximally. The OSC was synchronized by the MG1 to keep the quantization clock synchronized with the AMLL. For the following deep learning data recovery, a computer with a CPU core (Intel CORE i7-7700K) and two GPUs (NVidia GTX 1080ti) was programmed to construct linearization nets and matching nets. We used TensorFlow (v1.6) in Python as the framework to program the neural networks and LabVIEW to program the interfaces between the computer and instruments. To generate the training signals, another microwave generator (MG2, KEYSIGHT N5183B) was adopted. Controlled by the computer, it generated the signals to be sampled and input them into the MZM. Since the output signal of MG2 contained harmonics other than the standard sine, a series of custom-built low-pass filters (LPFs) were prepared for cancelling the harmonics to ensure that the output signal of MG2 was clean. To evaluate the performance of the ADC in untrained sine-like signal applications, we applied dual-tone signals and LFM signals as input to the ADC. The dual-tone signals were generated by combining MG2 and another microwave generator (MG3, Rhode and Schwarz SMA 100 A) and the LFM signals were generated via an arbitrary waveform generator (AWG, KEYSIGHT M8195A).

### Implementation of the deep neural networks

Inspired by image denoising, inpainting^16^, and superresolution^29,30^, the tasks of nonlinearity cancellation and mismatch compensation only require the neural networks to manipulate local data; they need not memorize the whole data sequence. Therefore, we could construct the neural networks to be purely convolutional, which has substantial advantages for the ADC application (e.g., immunity to data length variation and frequency spectrum aliasing). The neural networks were composed of the residual learning scheme^21^ and each linearization net was comprised of an input layer, two residual blocks, and an output layer. The input layer was a convolutional layer that converts one input channel to 32 feature channels, which is represented as follows:

$$Y_j = X_i \times W_{ij} + b_j, j = 1,2,...,32.$$

Input channel *X*_*i*_(*i*=1) consisted of an input data sequence that was convoluted with the *j*th convolution window *W*_*ij*_, whose window width was 3, in the “SAME” manner (padding the head and the tail of the input sequence with zeros such that the output is of the same length as the input). Then, we added the *j*th bias *b*_*j*_ to obtain the *j*th feature channel *Y*_*j*_. In the following residual blocks, two convolutional and activation layers were included. Each layer of convolution and activation was represented as follows:

$$Y_j = {\rm ReLU}\left(\mathop {\sum }\limits_{i = 1}^N X_i \times W_{ij} + b_j\right),j = 1,2,...,J.$$

In contrast to the input layer, this layer has a “ReLU” manipulation, namely, ReLU(*x*) = {0, *x*}. We changed the number of output feature channels *J* according to the pyramid structure^31^. At the end of each residual block, *J* *=* 34 or 38. As the output data of each residual block should be added to the input of the residual block but was unmatched in terms of the number of feature channels, we used an additional convolutional layer (with a window width of 1) to convert the number of channels of the input to the number of channels of the output^32^. The output layer was similar to the input layer of the calculation formula; however, it converted the 38 feature channels to one output data sequence. By adding the output data sequence to the original input data sequence^23,33^, the output of the linearization nets was obtained. For the matching nets, the original input data were several sequences from various quantization channels. Therefore, in the input layer of the matching nets, we conducted interleaving after individual convolutions as follows:

$$Y_j^m = X_i^m \times W_{ij}^m + b_j^m,j = 1,2,...,32,m = 1,2,$$

$$Y_j = \mathrm{ITL}(Y_j^1,Y_j^2).$$

The “ITL” manipulation is interleaving, namely, constructing the result sequence *Y*_*j*_ by alternately selecting the data in $Y_j^1$ and $Y_j^2$:

$$\begin{array}{ll}Y_j\left[ 1 \right],Y_j\left[ 2 \right],Y_j\left[ 3 \right],Y_j\left[ 4 \right],Y_j\left[ 5 \right]...\\ = Y_j^1[1],Y_j^2[1],Y_j^1[2],Y_j^2[2],Y_j^1[3]....\end{array}$$

For each input data sequence, we calculated 32 feature channels and used interleaving to construct 32 interleaved feature channels. The interleaved feature channels were double the length of the input data sequence. The following part of the “matching nets” was the same as that of the “linearization nets,” with two residual blocks and an output layer.

### Data acquisition, processing, and neural network training

For the standardized ADC performance characterizations^34^ and high-quality data acquisition, in the experimental demonstration, we use sine-waves for training and sine-like signals for the experimental validation. Based on the proposed analog-to-digital conversion architecture, 417 sine signals with various frequencies and amplitudes, dual-tone signals with various frequencies, and LFM signals with various frequencies and bandwidths were sampled using the experimental setup to construct the training dataset and the validation dataset. Since the sampling rate of the experimental setup was 20 GS/s, the frequencies of the sampled sine signals were randomly selected but uniformly distributed within the Nyquist bandwidth of 0–10 GHz. As the adopted real-time oscilloscope has a built-in bandwidth limit of 4.2 GHz, we discarded the frequencies from 4 to 6 GHz. By linking appropriate LPFs on the output of MG2, second-order or high-order harmonics of the output signals were eliminated. A LabVIEW program was developed for controlling MG2 to emit amplitude/frequency-varying signals. The amplitudes were also randomly selected and uniformly distributed within −2 to 15 dBm. The dual-tone signals were generated by the combination of MG2 and MG3, and the LFM signals were generated by AWG. Appropriate filters were also used in dual-tone and LFM signals to avoid harmonics in the generated signals. Data processing yielded the training set and the validation set by obtaining original/reference data pairs. To train the linearization nets, we regarded the distorted results as the original data and calculated the reference data for every distorted result. By removing the nonlinear harmonics via frequency-domain analysis and adding the harmonic power to the signal power, the processed signal was regarded as the reference data. The LFM signals whose spectra were not aliased were processed as such since frequency-domain analysis is inappropriate for aliased spectra. This data processing was performed using MATLAB codes. To train the matching nets, the original data were the reference data for the “linearization nets” that were obtained via the processing that is described above and the reference data were the recovered interleaved data. Frequency-domain manipulation was also used for reference data processing, removing channel mismatch distortions, and increasing the signal power. By selecting 367 data pairs as the training set and 50 data pairs as the validation set, we conducted neural network training by minimizing the loss in the training set.

$${\mathrm{Loss}}(\Theta ) = \frac{1}{L}\mathop {\sum }\limits_{l = 1}^L \left|Y_l^\Theta - Y_l^{\mathrm{REF}}\right|$$

We reconfigured the parameters of the neural networks $\Theta$ by adopting minimization algorithms to minimize the average absolute difference between the output of the neural networks $Y^\Theta$ and the reference data $Y^{REF}$. The minimization algorithm that was used in this work was adaptive gradient descent^35^ with backpropagation (the learning rate was 0.1 and decayed to 0.01 after 900 k epochs). Here, *L* represents the length of the data sequences, which is 1000 in the linearization nets and 2000 in the matching nets. Via several trials, the number of training epochs was fixed to 1 million for each neural network to ensure that the parameters had sufficiently converged and were not overfit. We calculated the loss in the validation set every 1000 epochs.

The deep neural networks in this work were trained with sine inputs and, consistent with “No Free Lunch” theory^36^ in machine learning, the trained networks were only applicable to sine-like waveforms. However, in future works, datasets with complicated waveforms could enable the neural networks to be applied in other application scenarios (this is discussed in detail in Suppl.).

### Simulation of the applicability of “matching nets” in multichannels

Using the experimental setup, the validity of the matching nets was demonstrated using two-channel data interleaving. For further sampling rate multiplication, we used the simulation results to demonstrate the performance of the matching nets in multichannel data interleaving. The simulation was conducted via the following steps:

1. Consider the reference data of the matching nets (calculated as in the “Materials and methods” section) as the reference data in the simulation. The original data will be calculated from the reference data by adding mismatch.
2. Divide the reference data into *N* channels (*N* varies from 2 to 8). This procedure is inverse to interleaving and allocates data into channels alternately.
3. Add channel mismatch to the data in each channel. The mismatch degree in the experimental setup is ~7 ps; therefore, the mismatch degrees in the simulations are randomly selected around 7 ps. This data processing can be implemented using MATLAB codes.
4. Use the artificially mismatched channels and reference data to train the matching nets for 500 k epochs and record the converged values.
5. Change the mismatch degrees and the number of channels *N* and repeat steps (2)–(4).

For each number of channels, ten mismatch degrees were considered and recorded (Fig. 4d).

### Supplementary experiment using MLL and a high-accuracy data acquisition board

To realize the high accuracy of the neural networks and demonstrate the potential of the proposed analog-to-digital conversion architecture in future high-dynamic high-accuracy applications, an ultralow-jitter MLL (Menlo Systems LAC-1550) was adopted to replace the AMLL and a high-accuracy electronic data acquisition board (Texas Instrument ADC16DX37EVM) replaced the OSC. The nominal timing jitter of the MLL was <2 fs and the ENOB of the data acquisition board was 9.37, thereby facilitating an ultralow noise floor. The repetition rate of the MLL was 100 MHz and the sampling rate of the data acquisition board was 100 MHz. Since the Nyquist bandwidth of the 100-MS/s ADC is 50 MHz, to acquire the training set and the validation set, we controlled the MG1 to generate signals from 400 to 450 MHz to match the passband of the low-pass filter, which could suppress the harmonics of signals from 330 to 500 MHz. The PD was replaced with a 300-MHz PD to avoid extra thermal noise. In total, 274 sine data were obtained, of which 244 were selected as the training set and 30 as the validation set. The data acquisition, processing, and neural network training methods were similar to those that are detailed in “Materials and methods”. After training, this setup was used to conduct subsampling of the 23.333-GHz signal.

### ENOB and SFDR characterizations

We conducted performance characterizations of our experimental setup using the IEEE standards. For an ADC system, single-tone (sine) signals are used for ENOB and SFDR characterizations.

When the signals to be sampled are of a single tone, ENOB can be represented by the ratio of the power of the signal to the power of all the noise and distortions as follows:

$$\begin{array}{lll}\mathrm{ENOB} &=& \frac{1}{{6.02}} \cdot \left( {10\log _{10}\left( {\frac{{P_{\mathrm{Signal}}}}{{P_{\mathrm{Noise}} + P_{\mathrm{Distortions}}}}} \right) - 1.76} \right)\\ &=& \frac{{\mathrm{SINAD - 1.76}}}{{6.02}}.\end{array}$$

Here, the SINAD was calculated in dB using the MATLAB “sinad()” function.

The SFDR of an ADC is defined as the ratio of the power of the signal to the power of the largest harmonic or distortion:

$${\rm SFDR}(dB) = {10\log} _{10}\left( \frac{P_{\rm Signal}}{P_{{\rm max}\_{\rm Harm}}\, or \,P_{{\rm max}\_{\rm Distortion}}} \right).$$

The powers of signals and harmonics or distortions are calculated from the spectra after the addition of a Blackman window.

### Evaluation of the neural network throughputs

Since linearization nets and matching nets have the same structure and hyperparameters, we only present the evaluation of linearization nets. First, we evaluated the experimental throughput based on dual GTX 1080ti GPUs. Because the neural networks were tolerant of data length variations, to avoid the margin latency of each iteration, we input massive data into the neural networks and calculated the average throughput of the neural networks. When the input data consisted of 128 × 8192 points and was iterated 1000 times, the average running time was 19.818 s and the corresponding throughput was 52.92 Mpts/s with 32-bit floating-point operations. Later, we theoretically evaluated the throughputs when linearization nets were implemented on various commercial deep learning accelerators. The complexity of the linearization nets was calculated as follows: every convolution with a width of 3 requires six floating-point operations and a ReLU activation requires one operation for each element. Therefore, if linearization nets output *N* points of data, the total number of required floating-point operations is

$$[6 \times (32 + 32 \times 34 \times 34 \times 34 \times 34 \times 38 \times 38 \times 38 \times 38) + 2 \times (34 + 38 + 32 \times 34 + 34 \times 38)] \times N = 35204 \times N.$$

The throughputs on six commercial deep learning accelerators, namely, Nvidia GTX 1080ti, Tesla P100, Tesla V100, Xilinx Alveo U200, Google TPU v2, and TPU v3, were evaluated according to the officially declared floating-point operations per second (FLOPS)^37–39^. The performances of the processers were characterized with various data types. For instance, the three processers of Nvidia provide 32-bit FLOPS and two of these (P100 and V100) provide 16-bit FLOPS. The Xilinx and Google processers only offer 16-bit FLOPS. The throughputs of the neural networks when running on these processers were calculated as

$$\mathrm{Throughput} = \frac{\mathrm{FLOPS}}{{35204}}({\mathrm{pts/s}}).$$

If the input data cache was assumed to be 256 kpts or 1 MB, the fps that is indicated in Fig. 5c was derived from the throughput results via the following formula:

$${\mathrm{fps}} = \frac{\mathrm{FLOPS}}{{256 \times 10^3}}({\mathrm{s}}^{ - 1}).$$

## Supplementary information

The clear version of the Supplementary Materials

## Data Availability

All data in this study can be obtained from the corresponding authors on reasonable request.
