# Supplementary material for: Deep-learning-powered photonic analog-to-digital conversion
Source: Light Sci Appl. 2019 Jul 17;8:66. doi: 10.1038/s41377-019-0176-4 (PMC6804794; doi:10.1038/s41377-019-0176-4)
Supplement: Supplementary file 1 — The clear version of the Supplementary Materials [file 41377_2019_176_MOESM1_ESM.docx]

# Supplementary Materials of

# Deep learning powered photonic analog-to-digital conversion

Shaofu Xu^1^, Xiuting Zou^1^, Bowen Ma^1^, Jianping Chen^1^, Lei Yu^1^, Weiwen Zou^1^*

^1^State Key Laboratory of Advanced Optical Communication Systems and Networks, Department of Electronic Engineering, Shanghai Jiao Tong University, Shanghai 200240, China.

*Correspondence to: [wzou@sjtu.edu.cn](mailto:wzou@sjtu.edu.cn)

**Supplementary discussions:**

**1. The neural networks immunity of input length variation.**

The proposed linearization nets and matching nets are all composed with convolutional layers. In neural networks, convolution is defined as the discrete way. The following schematic shows convolutions when input is 1-D sequence.

**Fig. S1 Convolutions with 1-D sequence.** The convolution window in the schematic is set at 3. The left subplot and the formula represent a single convolution and the right subplot represents the convolutions in multi-layer neural networks.

The output value is yielded by the dot-product of input segment and convolution window. So, the length needed of input segment is determined by the convolution window width. In the schematic, the window width is set as 3, so we can group every three input values to form an input segment. If we inspect the convolutions in multiple layers, an output value is only influenced by a segment from the input sequence. With limited number of convolutional layers (6 in our work), a final output value is influenced by a length-limited segment from the input sequence. In another word, the all-convolutional neural networks are trained to learn the local relations between the input segments and the corresponding output values. So, no matter how long is the input sequence, the all-convolutional neural networks would map input segments to the outputs correctly.

**2.** **Waveform generalization of the neural networks.**

An elemental theory in machine learning area, “No Free Lunch” theory^1^, declares that a learning machine trained with training set with finite examples could not universally applicable to the inference of infinite arbitrary testing examples. Consistently, in the DL-PADC, sine-wave trained neural networks will not be applicable to non-sine-alike complicated waveforms.

As the novel architecture combining deep learning and photonic ADC, we would better to follow a standardized performance characterization to show the ability of the proposed method. IEEE standards^2^ are set where ENOB and SFDR are characterized based on the digital results of sine input signals, so we apply sine-waves with the IEEE standards to characterize the DL-PADC performance in the experiment. Besides, to acquire a dataset which can reveal the defects of optical components themselves, we need to input high-quality signals with low spurs and low noise to the PADC system. Broadband complicated analog waveforms can be only provided by high-speed arbitrary waveform generators (AWGs), whereas the state-of-the-art commercial AWG performs ~ 6 ENOB (such as Keysight Ltd., M9502A^3^), which is not adequate to the high-quality dataset acquisition. Here, we apply a microwave source and filter group to generate high-quality sine signals.

Equivalent importantly, the generalization of complicated waveforms determines whether the method could be used in a broad range of applications. Limited by the state-of-the-art experimental condition, we use simulation results to reveal the potential feasibility of the proposed neural networks in complicated waveforms. In the simulation, we generate a group of complicated waveforms analogue to the echoes of ultra-wideband (UWB) radars^4^. As can be seen in Fig. S7a, the time domain waveform is fairly complicated. The details of these waveform generations can be summarized as follows. We assumed that a UWB radar transmits an LFM signal with bandwidth of 1.875 GHz and the signal was scattered back by an unknown object. Following the “multiple scattering center” target model of UWB radars, the echo of the unknown object can be regarded as the interference result of every scattering center on the object. Consequently, the echo waveforms can be super complicated in time domain. By Monte-Carlo simulation of the moving speed and scattering centers of the unknown objects, we got a dataset comprising 250 complicated waveforms. To simulate the distortion effect of the PADC system, we added nonlinearity of the MZM in the PADC system to the waveforms and then separated the waveforms to two mismatched channels. In the nonlinearization, we manipulated the waveform data by formula

$$y(t)=\sin(1.3\cdot x(t)+0.1)/1.3$$

The waveform $x(t)$ were normalized to 1 before applying to the formula and $y(t)$ represented the nonlinearized waveform due to the MZM. Then, the channel mismatch effect was simulated with similar method described in the fourth section in Method. The data were divided to 2 channels and then a mismatch of 7 ps was added to channel 2. After these distortion effects, we grouped the nonlinearized waveforms and their ideal references to be a dataset for linearization nets, and the mismatched waveforms and their ideal interleaved data were grouped as the dataset for matching nets. Totally, 250 distorted-reference data pairs were grouped for matching nets and 500 pairs are prepared for the linearization nets. Four fifth of these datasets were for training and one fifth were for validation.

As shown in Fig. S7b and c, the loss functions of linearization nets and matching nets dropped with training epochs and converged to a steady small value, implying the networks did not overfit and performed well on these complicated waveforms. The same effects in time domain and time-frequency domain were also revealed in Fig. S7d-i. Linearization nets could recover the nonlinearity and matching nets was effective in the mismatch recovery of complicated waveforms. Note that the neural networks and training method were the same with the proof-of-concept experiment. Therefore, there was no additional complexity introduced to this method rather than the dataset alteration. The simulation results could verify the potential waveform generalization and the broader applications of the proposed DL-PADC.

**3. Potential application discussion of DL-PADC**

ADC is the interface between the analog world and the digital computers and data storage, generally used in electronic information systems including communications, electronic measurement, radar etc. So, the potential applications of DL-PADC to high-frequency broadband RF field are highly expected. The PADC front-end provide a broadband signal reception and direct RF sampling (see refs. 3, 4), which is elegant to break through the traditional electronic bottleneck by taking advantage of the photonic technologies. Besides, the proposed architecture combines deep learning to solve the substantial defects of multi-channel PADC systems, improving the fidelity of quantized digital data.

In the proof-of-concept experiments, we adopt sine-waveforms to train the neural networks and reach high performances compared with state-of-the-art in-lab and commercial ADCs, validating the effectiveness of the neural networks on PADC system defects recovery. Consistent with the machine learning theory (as described in the second discussion), the sine-wave trained neural networks are not feasible in arbitrary complicated waveforms. Therefore, if the DL-PADC are to be used in the scenarios based on more complicated waveforms, dataset re-acquiring or augmentation based on complicated waveforms is needed.

Limited by the experimental conditions, we supply simulation results to reveal the potential of the proposed architecture on complicated waveforms by dataset alteration. Details of the simulation are described in the second discussion. The results infer that with the further development of the datasets based on complicated waveforms, the application area of this architecture could be broadened, including ultra-wideband radar, high-resolution microwave imaging, and other high-frequency broadband applications.

**Reference:**

1. D. H. Wolpert and W. G. Macready, No free lunch theorem for optimization, IEEE Trans. Evolutionary Computing 1, 67-82 (1997).
2. IEEE, IEEE standard for terminology and test methods for analog-to-digital converters, http://ieeexplore.ieee.org/xpl/articleDetails.jsp?arnumber=929859&contentType=Standards.
3. Keysight Technology, M8195A 65 GSa/s Arbitrary Waveform Generator Data Sheet, <http://literature.cdn.keysight.com/litweb/pdf/5992-0014EN.pdf>.
4. M. I. Skolnik, *Radar Handbook (3^rd^ edition)*, by Mc Graw Hill, (2008).


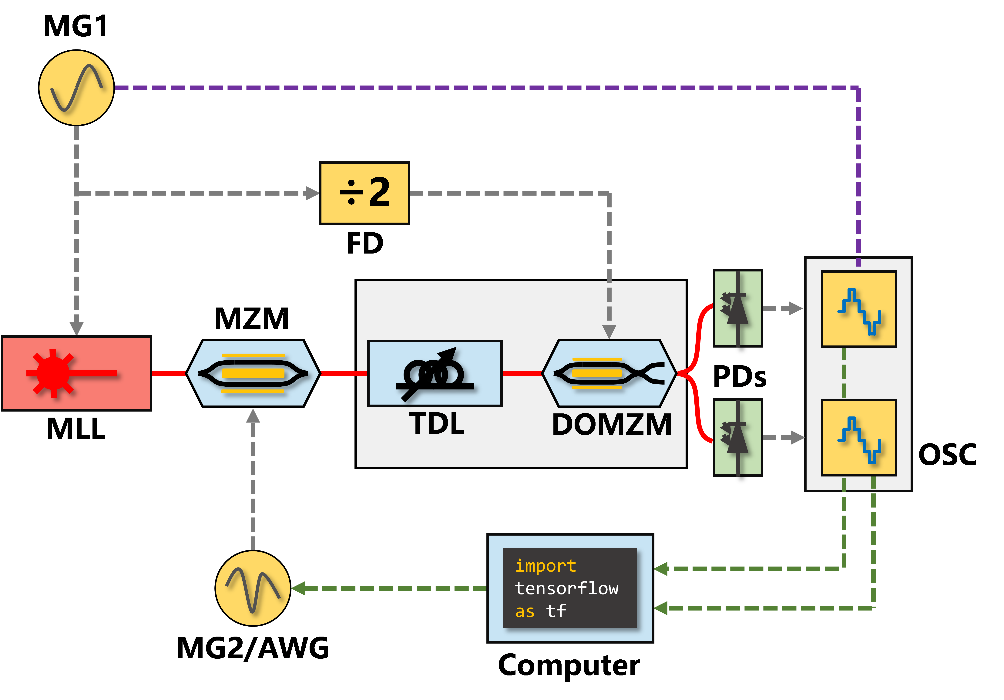


**Fig. S2. Experimental setup of the 20-GS/s photonic ADC**. MG, microwave generator; FD, frequency divider; MLL, mode-locked laser; MZM, Mach–Zehnder modulator; TDL, tunable delay line; DOMZM, dual-output Mach–Zehnder modulator; PD, photon detector; OSC, oscilloscope; AWG, arbitrary waveform generator.

**Fig. S3. Sine signal results before and after linearization nets.** These data are randomly chosen in the validation set. Gray dashed curve is the original sampled and quantized data. Blue solid curve indicates the recovered data after linearization nets.

**Fig. S4. Dual-tone and LFM signal results before and after linearization nets.** In all frequency spectra, gray dashed curves represent data before linearization, and blue solid curves denote recovered data after linearization nets. STFT plots are also given for LFM signals; the left and right columns are data before and after the linearization nets, respectively.

**Fig. S5. Sine signal results before and after the matching nets.** These data are randomly chosen in the validation set. Gray dashed curve is the mismatched interleaved data. Blue solid curve is the recovered data after matching nets.

**Fig. S6. LFM signal results before and after the matching nets.** In all frequency spectra, gray dashed curves represent data before the matching nets, and blue solid curves denote the recovered data after the matching nets. STFT plots are also given for LFM signals; the left and right columns are data before and after the matching nets, respectively.

**Fig. S7. Simulation results of the neural networks to complicated waveforms. a,** An example of the simulated waveforms of UWB radar echoes in time domain. **b** and **c,** Loss functions descending with train epoch growth, corresponding to the linearization nets and matching nets, respectively. Note that the training loss overlaps with validation loss so the training loss seems invisible. **d** and **e,** Signal recovery effect of linearization nets and matching nets in time domain. **f-i,** Signal recovery effect of linearization nets and matching nets in time-frequency domain, where upper row is the distorted signals and the lower row is the recovered signals.
